# Crystal structure of bis­{2-[bis­(2-hy­droxy­eth­yl)amino]­ethanol-κ^4^ *O*,*N*,*O*′,*O*′′}cadmium terephthalate

**DOI:** 10.1107/S1600536814022375

**Published:** 2014-10-18

**Authors:** Ya-Ping Li, Li-Ying Han, Julia Ming, Guan-Fang Su

**Affiliations:** a Department of Ophthalmology, The Second Hospital of Jilin University, 218 Ziqiang Street, Changchun 130041, People’s Republic of China; b Department of Gynaecology, The Second Hospital of Jilin University, 218 Ziqiang Street, Changchun 130041, People’s Republic of China; c St Erik’s Eye Hospital, Karolinska Institutet, Polhemsgatan 50, SE-112 82 Stockholm, Sweden

**Keywords:** crystal structure, cadmium complex, terephthalate, hydrogen bonding

## Abstract

In the title salt, [Cd(C_6_H_15_NO_3_)_2_](C_8_H_4_O_4_), the Cd^2+^ cation is coordinated by six O atoms and two N atoms from two tetra­dentate 2-[bis­(2-hy­droxy­eth­yl)amino]­ethanol ligands, displaying a distorted square-anti­prismatic coordination. The terephthalate dianion does not coordinate to the cation but is connected through O⋯H—O hydrogen bonds of medium strength to the complex cations, leading to a layered structure extending parallel to (100).

## Related literature   

For Cd—O and Cd—N bond lengths resulting from CdN_2_O_6_ and CdN_4_O_4_ coordination sets, see: Shirvan & Dezfuli (2012 ▶); Shi & Tiekink (2009 ▶).
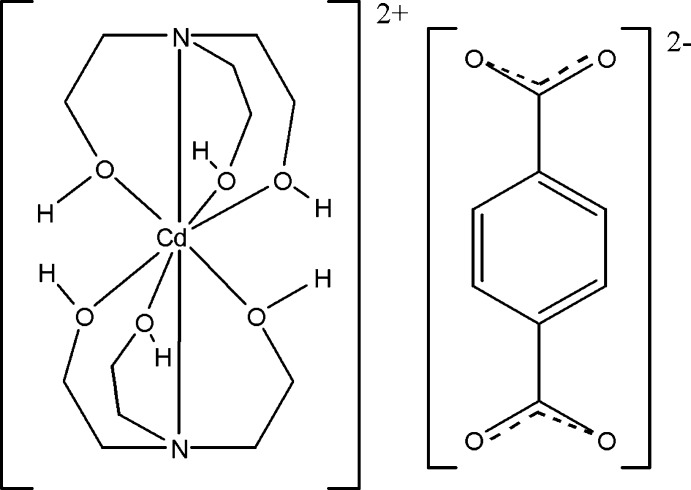


## Experimental   

### Crystal data  

[Cd(C_6_H_15_NO_3_)_2_](C_8_H_4_O_4_)
*M* *_r_* = 574.89
Orthorhombic, 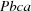

*a* = 13.2789 (12) Å
*b* = 14.6329 (14) Å
*c* = 24.278 (2) Å
*V* = 4717.4 (8) Å^3^
*Z* = 8
Mo *K*α radiation
μ = 0.98 mm^−1^
*T* = 296 K
0.28 × 0.25 × 0.24 mm

### Data collection  

Bruker APEXII CCD diffractometer
Absorption correction: multi-scan (*SADABS*; Bruker, 2002 ▶) *T*_min_ = 0.725, *T*_max_ = 0.812
28314 measured reflections
4639 independent reflections
2694 reflections with *I* > 2σ(*I*)
*R*_int_ = 0.093

### Refinement  

*R*[*F* ^2^ > 2σ(*F* ^2^)] = 0.037
*wR*(*F* ^2^) = 0.099
*S* = 0.98
4639 reflections
316 parameters
6 restraints
H atoms treated by a mixture of independent and constrained refinement
Δρ_max_ = 0.77 e Å^−3^
Δρ_min_ = −0.50 e Å^−3^

### 

Data collection: *APEX2* (Bruker, 2002 ▶); cell refinement: *SAINT* (Bruker, 2002 ▶); data reduction: *SAINT*; program(s) used to solve structure: *SHELXS97* (Sheldrick, 2008 ▶); program(s) used to refine structure: *SHELXL97* (Sheldrick, 2008 ▶); molecular graphics: *SHELXTL97* (Sheldrick, 2008 ▶); software used to prepare material for publication: *SHELXTL* and *publCIF* (Westrip, 2010 ▶).

## Supplementary Material

Crystal structure: contains datablock(s) global, I. DOI: 10.1107/S1600536814022375/wm5070sup1.cif

Structure factors: contains datablock(s) I. DOI: 10.1107/S1600536814022375/wm5070Isup2.hkl

**Fig. 1.**
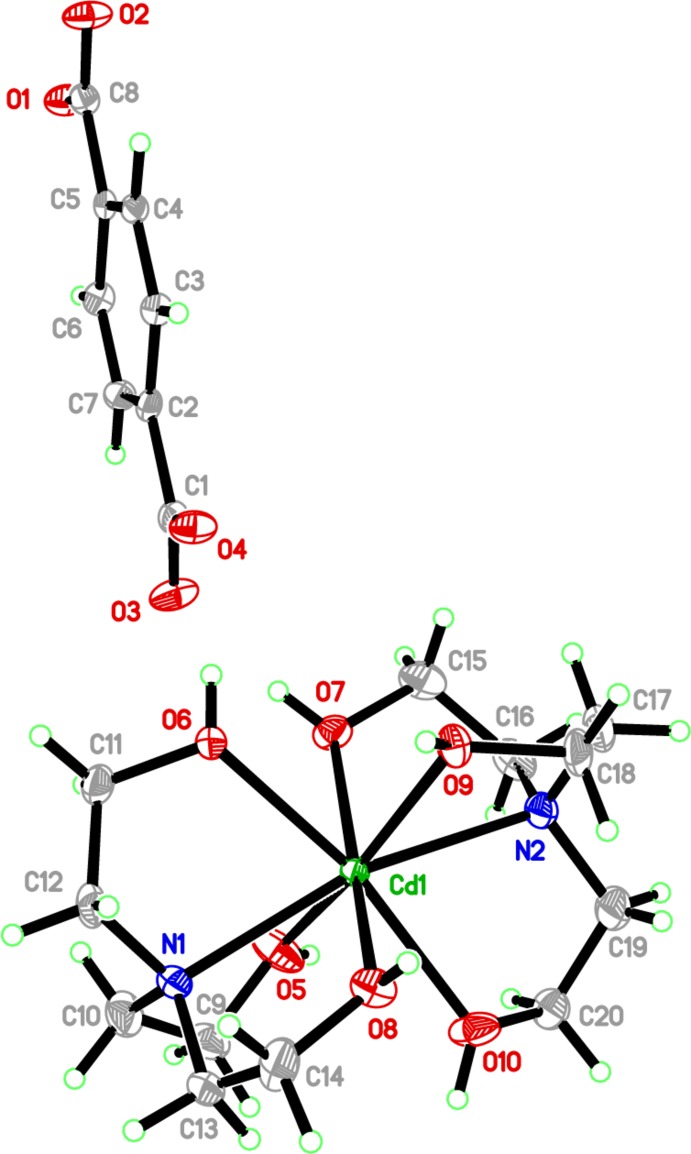
The molecular components of the title compound with displacement ellipsoids drawn at the 30% probability level.

. DOI: 10.1107/S1600536814022375/wm5070fig1.tif
The mol­ecular components of the title compound with displacement ellipsoids drawn at the 30% probability level.

**Fig. 2.**
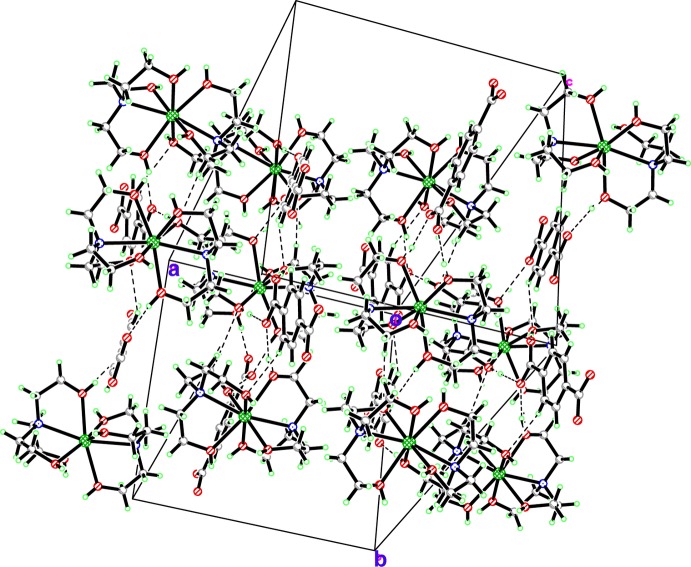
The packing of the molecular components in the crystal structure of the title compound. O—H···O hydrogen bonds are indicated by dashed lines.

. DOI: 10.1107/S1600536814022375/wm5070fig2.tif
The packing of the mol­ecular components in the crystal structure of the title compound. O—H⋯O hydrogen bonds are indicated by dashed lines.

CCDC reference: 1028647

Additional supporting information:  crystallographic information; 3D view; checkCIF report

## Figures and Tables

**Table 1:** Hydrogen-bond geometry (, )

*D*H*A*	*D*H	H*A*	*D* *A*	*D*H*A*
O6H01O4	0.84(2)	1.86(2)	2.692(4)	168(5)
O8H02O2^i^	0.83(2)	1.79(2)	2.612(4)	170(5)
O5H03O4^ii^	0.84(2)	1.82(2)	2.645(5)	170(6)
O9H04O1^i^	0.84(2)	1.84(2)	2.673(4)	169(5)
O10H05O1^iii^	0.84(2)	1.82(2)	2.647(4)	169(6)
O7H06O3	0.86(2)	1.78(2)	2.635(4)	171(5)

Symmetry codes: (i) 
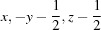
; (ii) 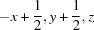; (iii) 
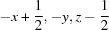
.

## supplementary crystallographic information

### S1. Preparation

The synthesis was performed under hydro­thermal conditions. A mixture of Cd(CH_3_COO)_2_·2H_2_O, (0.2 mmol, 0.053 g), [tris­(2-hy­droxy­ethyl)­amino]­ethanol (0.4 mmol, 0.062 g), sodium terephthalate (0.2 mmol, 0.042 g) and water (20 ml) in a 30 ml stainless steel reactor with a Teflon liner was heated from 293 to 433 K in 2 h and a constant temperature was maintained at 433 K for 72 h, after which the mixture was cooled to 298 K. Colorless crystals of the title compound were recovered from the resulting reaction solution.

### S2. Refinement

The C—H H atoms were positioned with idealized geometry and refined with *U*_iso_(H) = 1.2*U*_eq_(C) using a riding model. The hy­droxy H-atoms were located in a difference Fourier map and were refined with an O—H distance restrained to 0.85 (1) Å and with *U*_iso_(H) = 1.5*U*_eq_(O). One reflection (002) was obstracted from the beamstop and was omitted from the refinement.

### Crystal data

**Table:**

[Cd(C_6_H_15_NO_3_)_2_](C_8_H_4_O_4_)	*F*(000) = 2368
*M**_r_* = 574.89	*D*_x_ = 1.619 Mg m^−^^3^
Orthorhombic, *P**b**c**a*	Mo *K*α radiation, λ = 0.71073 Å
Hall symbol: -P 2ac 2ab	Cell parameters from 4657 reflections
*a* = 13.2789 (12) Å	θ = 1.7–22.8°
*b* = 14.6329 (14) Å	µ = 0.98 mm^−^^1^
*c* = 24.278 (2) Å	*T* = 296 K
*V* = 4717.4 (8) Å^3^	Block, colorless
*Z* = 8	0.28 × 0.25 × 0.24 mm

### Data collection

**Table:**

Bruker APEXII CCD diffractometer	4639 independent reflections
Radiation source: fine-focus sealed tube	2694 reflections with *I* > 2σ(*I*)
Graphite monochromator	*R*_int_ = 0.093
φ and ω scans	θ_max_ = 26.0°, θ_min_ = 2.2°
Absorption correction: multi-scan (*SADABS*; Bruker, 2002)	*h* = −16→15
*T*_min_ = 0.725, *T*_max_ = 0.812	*k* = −17→18
28314 measured reflections	*l* = −29→29

### Refinement

**Table:**

Refinement on *F*^2^	Primary atom site location: structure-invariant direct methods
Least-squares matrix: full	Secondary atom site location: difference Fourier map
*R*[*F*^2^ > 2σ(*F*^2^)] = 0.037	Hydrogen site location: inferred from neighbouring sites
*wR*(*F*^2^) = 0.099	H atoms treated by a mixture of independent and constrained refinement
*S* = 0.98	*w* = 1/[σ^2^(*F*_o_^2^) + (0.0439*P*)^2^ + 0.0385*P*] where *P* = (*F*_o_^2^ + 2*F*_c_^2^)/3
4639 reflections	(Δ/σ)_max_ = 0.001
316 parameters	Δρ_max_ = 0.77 e Å^−^^3^
6 restraints	Δρ_min_ = −0.50 e Å^−^^3^

### Special details

**Table:**

Geometry. All esds (except the esd in the dihedral angle between two l.s. planes) are estimated using the full covariance matrix. The cell esds are taken into account individually in the estimation of esds in distances, angles and torsion angles; correlations between esds in cell parameters are only used when they are defined by crystal symmetry. An approximate (isotropic) treatment of cell esds is used for estimating esds involving l.s. planes.
Refinement. Refinement of F^2^ against ALL reflections. The weighted R-factor wR and goodness of fit S are based on F^2^, conventional R-factors R are based on F, with F set to zero for negative F^2^. The threshold expression of F^2^ > 2sigma(F^2^) is used only for calculating R-factors(gt) etc. and is not relevant to the choice of reflections for refinement. R-factors based on F^2^ are statistically about twice as large as those based on F, and R- factors based on ALL data will be even larger.

### Fractional atomic coordinates and isotropic or equivalent isotropic displacement parameters (Å^2^)

**Table:**

	*x*	*y*	*z*	*U*_iso_*/*U*_eq_	
C1	0.2024 (3)	−0.1259 (3)	0.77515 (18)	0.0269 (10)	
C2	0.2153 (3)	−0.1917 (3)	0.82266 (18)	0.0229 (10)	
C3	0.2323 (3)	−0.2840 (3)	0.81455 (17)	0.0232 (10)	
H3	0.2355	−0.3067	0.7788	0.028*	
C4	0.2447 (3)	−0.3434 (3)	0.85847 (17)	0.0214 (8)	
H4	0.2562	−0.4052	0.8520	0.026*	
C5	0.2400 (3)	−0.3108 (3)	0.91230 (18)	0.0216 (9)	
C6	0.2230 (3)	−0.2185 (3)	0.92059 (18)	0.0251 (10)	
H6	0.2196	−0.1959	0.9563	0.030*	
C7	0.2110 (3)	−0.1594 (3)	0.87684 (17)	0.0274 (10)	
H7	0.1999	−0.0976	0.8834	0.033*	
C8	0.2551 (3)	−0.3762 (3)	0.95972 (16)	0.0250 (9)	
C9	0.1119 (4)	0.2604 (3)	0.6575 (2)	0.0433 (13)	
H9A	0.1278	0.2958	0.6248	0.052*	
H9B	0.0880	0.3017	0.6859	0.052*	
C10	0.0344 (4)	0.1908 (3)	0.6448 (2)	0.0474 (14)	
H10A	0.0152	0.1600	0.6786	0.057*	
H10B	−0.0249	0.2210	0.6303	0.057*	
C11	0.0503 (3)	−0.0201 (3)	0.65781 (19)	0.0338 (12)	
H11A	0.0488	0.0142	0.6920	0.041*	
H11B	0.0093	−0.0745	0.6624	0.041*	
C12	0.0110 (3)	0.0362 (3)	0.6121 (2)	0.0366 (13)	
H12A	−0.0589	0.0512	0.6194	0.044*	
H12B	0.0136	0.0011	0.5783	0.044*	
C13	0.0602 (4)	0.1576 (4)	0.5480 (2)	0.0496 (15)	
H13A	0.0903	0.2179	0.5463	0.060*	
H13B	−0.0107	0.1640	0.5391	0.060*	
C14	0.1083 (4)	0.0988 (4)	0.5058 (2)	0.0497 (15)	
H14A	0.0694	0.0432	0.5011	0.060*	
H14B	0.1102	0.1306	0.4708	0.060*	
C15	0.3955 (4)	0.0473 (4)	0.7273 (2)	0.0451 (14)	
H15A	0.4191	−0.0147	0.7216	0.054*	
H15B	0.4024	0.0620	0.7661	0.054*	
C16	0.4576 (4)	0.1130 (4)	0.6931 (2)	0.0430 (13)	
H16A	0.4396	0.1752	0.7028	0.052*	
H16B	0.5283	0.1044	0.7016	0.052*	
C17	0.4911 (4)	0.0136 (3)	0.6150 (2)	0.0437 (14)	
H17A	0.5602	0.0266	0.6047	0.052*	
H17B	0.4922	−0.0301	0.6450	0.052*	
C18	0.4374 (3)	−0.0270 (3)	0.5672 (2)	0.0416 (13)	
H18A	0.4369	0.0155	0.5365	0.050*	
H18B	0.4705	−0.0828	0.5555	0.050*	
C19	0.4816 (4)	0.1776 (3)	0.6017 (2)	0.0502 (15)	
H19A	0.5417	0.2007	0.6197	0.060*	
H19B	0.5008	0.1565	0.5653	0.060*	
C20	0.4083 (3)	0.2523 (3)	0.5961 (2)	0.0408 (12)	
H20A	0.3934	0.2782	0.6319	0.049*	
H20B	0.4355	0.3001	0.5728	0.049*	
N1	0.0693 (3)	0.1224 (3)	0.60478 (15)	0.0306 (9)	
N2	0.4415 (3)	0.0993 (3)	0.63382 (16)	0.0326 (9)	
O1	0.2571 (2)	−0.3443 (2)	1.00821 (12)	0.0369 (8)	
O2	0.2658 (2)	−0.4589 (2)	0.94888 (12)	0.0398 (9)	
O3	0.1960 (3)	−0.0423 (2)	0.78535 (12)	0.0408 (9)	
O4	0.1988 (3)	−0.1589 (2)	0.72712 (12)	0.0346 (8)	
O5	0.1974 (3)	0.2125 (2)	0.67582 (16)	0.0522 (10)	
H03	0.236 (4)	0.251 (3)	0.690 (2)	0.078*	
O6	0.1506 (2)	−0.0445 (2)	0.64437 (12)	0.0270 (7)	
H01	0.174 (3)	−0.080 (3)	0.6685 (15)	0.040*	
O7	0.2943 (3)	0.0547 (2)	0.71137 (12)	0.0353 (8)	
H06	0.257 (3)	0.026 (3)	0.7342 (17)	0.053*	
O8	0.2064 (3)	0.0772 (2)	0.52263 (13)	0.0342 (8)	
H02	0.226 (3)	0.035 (3)	0.5023 (18)	0.051*	
O9	0.3366 (2)	−0.0460 (2)	0.58471 (12)	0.0314 (8)	
H04	0.304 (3)	−0.078 (3)	0.5619 (16)	0.047*	
O10	0.3200 (3)	0.2158 (2)	0.57235 (16)	0.0534 (11)	
H05	0.289 (4)	0.255 (3)	0.554 (2)	0.080*	
Cd1	0.25186 (2)	0.085556 (19)	0.618765 (11)	0.02039 (10)	

### Atomic displacement parameters (Å^2^)

**Table:**

	*U*^11^	*U*^22^	*U*^33^	*U*^12^	*U*^13^	*U*^23^
C1	0.024 (2)	0.034 (3)	0.023 (3)	0.002 (2)	0.004 (2)	0.003 (2)
C2	0.019 (2)	0.027 (3)	0.022 (2)	−0.0035 (17)	0.0035 (18)	0.0032 (19)
C3	0.025 (3)	0.027 (2)	0.017 (2)	−0.0013 (18)	−0.0014 (18)	−0.0037 (18)
C4	0.0197 (19)	0.020 (2)	0.025 (2)	−0.001 (2)	0.000 (2)	−0.0003 (18)
C5	0.015 (2)	0.027 (2)	0.022 (2)	−0.0026 (19)	0.003 (2)	−0.0009 (17)
C6	0.031 (3)	0.028 (3)	0.016 (2)	−0.0021 (18)	0.0010 (18)	−0.0038 (18)
C7	0.029 (2)	0.023 (2)	0.031 (3)	0.0015 (19)	0.004 (2)	−0.004 (2)
C8	0.024 (2)	0.030 (2)	0.021 (2)	0.003 (2)	0.000 (2)	0.0019 (19)
C9	0.042 (3)	0.031 (3)	0.057 (4)	0.008 (2)	−0.005 (3)	0.000 (3)
C10	0.036 (3)	0.034 (3)	0.073 (4)	0.005 (2)	0.012 (3)	−0.005 (3)
C11	0.030 (3)	0.037 (3)	0.035 (3)	−0.002 (2)	0.012 (2)	0.001 (2)
C12	0.019 (2)	0.042 (3)	0.049 (3)	−0.006 (2)	0.000 (2)	−0.002 (3)
C13	0.028 (3)	0.069 (4)	0.052 (4)	0.009 (3)	−0.002 (3)	0.032 (3)
C14	0.034 (3)	0.086 (4)	0.029 (3)	−0.017 (3)	−0.007 (2)	0.008 (3)
C15	0.051 (3)	0.053 (3)	0.032 (3)	0.008 (3)	−0.016 (3)	0.001 (3)
C16	0.036 (3)	0.046 (3)	0.047 (3)	0.002 (2)	−0.019 (3)	−0.008 (3)
C17	0.024 (3)	0.046 (3)	0.062 (4)	0.002 (2)	0.009 (2)	−0.003 (3)
C18	0.029 (3)	0.049 (3)	0.047 (3)	0.004 (2)	0.019 (2)	−0.003 (3)
C19	0.033 (3)	0.041 (3)	0.077 (4)	−0.008 (2)	0.007 (3)	0.007 (3)
C20	0.034 (3)	0.032 (3)	0.056 (3)	−0.008 (2)	−0.003 (2)	0.000 (3)
N1	0.028 (2)	0.031 (2)	0.033 (2)	0.0069 (17)	0.0026 (18)	0.0043 (18)
N2	0.024 (2)	0.031 (2)	0.043 (3)	−0.0033 (17)	−0.0015 (18)	0.0016 (19)
O1	0.059 (2)	0.0299 (17)	0.0212 (16)	0.0052 (17)	0.0000 (17)	0.0011 (13)
O2	0.073 (3)	0.0216 (17)	0.0246 (17)	0.0099 (17)	0.0092 (16)	0.0015 (14)
O3	0.071 (2)	0.0246 (19)	0.0265 (19)	0.0041 (17)	0.0081 (17)	0.0015 (15)
O4	0.052 (2)	0.0309 (19)	0.0208 (17)	0.0054 (16)	−0.0057 (16)	0.0014 (15)
O5	0.049 (2)	0.046 (2)	0.061 (3)	0.0186 (19)	−0.025 (2)	−0.019 (2)
O6	0.0220 (17)	0.0293 (18)	0.0296 (19)	0.0016 (13)	0.0023 (14)	0.0040 (15)
O7	0.0362 (19)	0.041 (2)	0.029 (2)	−0.0052 (16)	−0.0096 (15)	0.0083 (16)
O8	0.0411 (19)	0.035 (2)	0.0263 (19)	0.0046 (16)	−0.0084 (16)	−0.0061 (15)
O9	0.0274 (19)	0.039 (2)	0.0273 (19)	0.0003 (15)	0.0012 (14)	−0.0075 (15)
O10	0.049 (2)	0.046 (2)	0.065 (3)	−0.0230 (19)	−0.030 (2)	0.0273 (19)
Cd1	0.01904 (16)	0.02366 (17)	0.01848 (16)	−0.00051 (15)	−0.00175 (15)	0.00114 (13)

### Geometric parameters (Å, º)

**Table:**

C1—O3	1.250 (5)	C14—H14B	0.9700
C1—O4	1.263 (5)	C15—O7	1.402 (5)
C1—C2	1.512 (6)	C15—C16	1.513 (7)
C2—C3	1.385 (5)	C15—H15A	0.9700
C2—C7	1.398 (6)	C15—H15B	0.9700
C3—C4	1.385 (5)	C16—N2	1.469 (6)
C3—H3	0.9300	C16—H16A	0.9700
C4—C5	1.393 (6)	C16—H16B	0.9700
C4—H4	0.9300	C17—C18	1.486 (6)
C5—C6	1.384 (5)	C17—N2	1.488 (5)
C5—C8	1.511 (6)	C17—H17A	0.9700
C6—C7	1.378 (6)	C17—H17B	0.9700
C6—H6	0.9300	C18—O9	1.432 (5)
C7—H7	0.9300	C18—H18A	0.9700
C8—O2	1.246 (5)	C18—H18B	0.9700
C8—O1	1.267 (5)	C19—C20	1.469 (6)
C9—O5	1.407 (5)	C19—N2	1.485 (6)
C9—C10	1.480 (6)	C19—H19A	0.9700
C9—H9A	0.9700	C19—H19B	0.9700
C9—H9B	0.9700	C20—O10	1.412 (5)
C10—N1	1.470 (6)	C20—H20A	0.9700
C10—H10A	0.9700	C20—H20B	0.9700
C10—H10B	0.9700	N1—Cd1	2.506 (4)
C11—O6	1.417 (5)	N2—Cd1	2.553 (4)
C11—C12	1.477 (6)	O5—Cd1	2.427 (3)
C11—H11A	0.9700	O5—H03	0.84 (2)
C11—H11B	0.9700	O6—Cd1	2.412 (3)
C12—N1	1.490 (6)	O6—H01	0.841 (19)
C12—H12A	0.9700	O7—Cd1	2.362 (3)
C12—H12B	0.9700	O7—H06	0.859 (19)
C13—N1	1.477 (6)	O8—Cd1	2.414 (3)
C13—C14	1.481 (7)	O8—H02	0.831 (19)
C13—H13A	0.9700	O9—Cd1	2.378 (3)
C13—H13B	0.9700	O9—H04	0.841 (19)
C14—O8	1.402 (6)	O10—Cd1	2.392 (3)
C14—H14A	0.9700	O10—H05	0.84 (2)
			
O3—C1—O4	123.7 (4)	H17A—C17—H17B	108.0
O3—C1—C2	118.6 (4)	O9—C18—C17	107.1 (4)
O4—C1—C2	117.7 (4)	O9—C18—H18A	110.3
C3—C2—C7	118.0 (4)	C17—C18—H18A	110.3
C3—C2—C1	122.1 (4)	O9—C18—H18B	110.3
C7—C2—C1	119.9 (4)	C17—C18—H18B	110.3
C4—C3—C2	121.5 (4)	H18A—C18—H18B	108.5
C4—C3—H3	119.3	C20—C19—N2	112.6 (4)
C2—C3—H3	119.3	C20—C19—H19A	109.1
C3—C4—C5	120.1 (4)	N2—C19—H19A	109.1
C3—C4—H4	119.9	C20—C19—H19B	109.1
C5—C4—H4	119.9	N2—C19—H19B	109.1
C6—C5—C4	118.6 (4)	H19A—C19—H19B	107.8
C6—C5—C8	122.0 (4)	O10—C20—C19	107.9 (4)
C4—C5—C8	119.4 (4)	O10—C20—H20A	110.1
C7—C6—C5	121.2 (4)	C19—C20—H20A	110.1
C7—C6—H6	119.4	O10—C20—H20B	110.1
C5—C6—H6	119.4	C19—C20—H20B	110.1
C6—C7—C2	120.6 (4)	H20A—C20—H20B	108.4
C6—C7—H7	119.7	C10—N1—C13	110.7 (4)
C2—C7—H7	119.7	C10—N1—C12	109.5 (4)
O2—C8—O1	123.5 (4)	C13—N1—C12	111.4 (4)
O2—C8—C5	118.0 (4)	C10—N1—Cd1	111.2 (3)
O1—C8—C5	118.4 (4)	C13—N1—Cd1	106.3 (3)
O5—C9—C10	106.5 (4)	C12—N1—Cd1	107.7 (3)
O5—C9—H9A	110.4	C16—N2—C19	111.0 (4)
C10—C9—H9A	110.4	C16—N2—C17	110.6 (4)
O5—C9—H9B	110.4	C19—N2—C17	109.3 (4)
C10—C9—H9B	110.4	C16—N2—Cd1	107.1 (3)
H9A—C9—H9B	108.6	C19—N2—Cd1	109.8 (3)
N1—C10—C9	112.8 (4)	C17—N2—Cd1	109.0 (3)
N1—C10—H10A	109.0	C9—O5—Cd1	116.2 (3)
C9—C10—H10A	109.0	C9—O5—H03	107 (4)
N1—C10—H10B	109.0	Cd1—O5—H03	124 (4)
C9—C10—H10B	109.0	C11—O6—Cd1	112.6 (2)
H10A—C10—H10B	107.8	C11—O6—H01	110 (3)
O6—C11—C12	107.4 (3)	Cd1—O6—H01	117 (3)
O6—C11—H11A	110.2	C15—O7—Cd1	120.4 (3)
C12—C11—H11A	110.2	C15—O7—H06	110 (3)
O6—C11—H11B	110.2	Cd1—O7—H06	125 (3)
C12—C11—H11B	110.2	C14—O8—Cd1	120.2 (3)
H11A—C11—H11B	108.5	C14—O8—H02	107 (3)
C11—C12—N1	112.3 (4)	Cd1—O8—H02	122 (4)
C11—C12—H12A	109.1	C18—O9—Cd1	112.8 (3)
N1—C12—H12A	109.1	C18—O9—H04	113 (4)
C11—C12—H12B	109.1	Cd1—O9—H04	116 (4)
N1—C12—H12B	109.1	C20—O10—Cd1	115.0 (3)
H12A—C12—H12B	107.9	C20—O10—H05	112 (4)
N1—C13—C14	114.0 (4)	Cd1—O10—H05	127 (4)
N1—C13—H13A	108.8	O7—Cd1—O9	93.62 (11)
C14—C13—H13A	108.8	O7—Cd1—O10	120.72 (11)
N1—C13—H13B	108.8	O9—Cd1—O10	107.59 (13)
C14—C13—H13B	108.8	O7—Cd1—O6	74.73 (10)
H13A—C13—H13B	107.6	O9—Cd1—O6	73.42 (10)
O8—C14—C13	109.4 (4)	O10—Cd1—O6	163.97 (10)
O8—C14—H14A	109.8	O7—Cd1—O8	166.04 (11)
C13—C14—H14A	109.8	O9—Cd1—O8	74.99 (10)
O8—C14—H14B	109.8	O10—Cd1—O8	71.32 (12)
C13—C14—H14B	109.8	O6—Cd1—O8	93.99 (11)
H14A—C14—H14B	108.3	O7—Cd1—O5	71.00 (12)
O7—C15—C16	108.8 (4)	O9—Cd1—O5	163.50 (11)
O7—C15—H15A	109.9	O10—Cd1—O5	76.81 (14)
C16—C15—H15A	109.9	O6—Cd1—O5	106.89 (12)
O7—C15—H15B	109.9	O8—Cd1—O5	121.08 (11)
C16—C15—H15B	109.9	O7—Cd1—N1	113.65 (12)
H15A—C15—H15B	108.3	O9—Cd1—N1	125.77 (11)
N2—C16—C15	111.8 (4)	O10—Cd1—N1	97.51 (12)
N2—C16—H16A	109.3	O6—Cd1—N1	70.45 (11)
C15—C16—H16A	109.3	O8—Cd1—N1	68.78 (12)
N2—C16—H16B	109.3	O5—Cd1—N1	67.95 (12)
C15—C16—H16B	109.3	O7—Cd1—N2	69.10 (12)
H16A—C16—H16B	107.9	O9—Cd1—N2	69.32 (11)
C18—C17—N2	111.4 (4)	O10—Cd1—N2	68.38 (12)
C18—C17—H17A	109.4	O6—Cd1—N2	125.13 (11)
N2—C17—H17A	109.4	O8—Cd1—N2	112.90 (12)
C18—C17—H17B	109.4	O5—Cd1—N2	98.74 (12)
N2—C17—H17B	109.4	N1—Cd1—N2	163.06 (13)
			
O3—C1—C2—C3	−173.8 (4)	C20—O10—Cd1—O5	78.4 (3)
O4—C1—C2—C3	6.0 (6)	C20—O10—Cd1—N1	143.5 (3)
O3—C1—C2—C7	5.6 (6)	C20—O10—Cd1—N2	−26.8 (3)
O4—C1—C2—C7	−174.5 (4)	C11—O6—Cd1—O7	100.0 (3)
C7—C2—C3—C4	0.1 (6)	C11—O6—Cd1—O9	−161.4 (3)
C1—C2—C3—C4	179.5 (4)	C11—O6—Cd1—O10	−65.3 (5)
C2—C3—C4—C5	0.1 (6)	C11—O6—Cd1—O8	−88.4 (3)
C3—C4—C5—C6	−0.1 (6)	C11—O6—Cd1—O5	35.8 (3)
C3—C4—C5—C8	−179.2 (4)	C11—O6—Cd1—N1	−22.5 (3)
C4—C5—C6—C7	−0.1 (6)	C11—O6—Cd1—N2	149.9 (3)
C8—C5—C6—C7	179.0 (4)	C14—O8—Cd1—O7	105.2 (5)
C5—C6—C7—C2	0.3 (7)	C14—O8—Cd1—O9	141.3 (4)
C3—C2—C7—C6	−0.3 (7)	C14—O8—Cd1—O10	−103.8 (4)
C1—C2—C7—C6	−179.8 (4)	C14—O8—Cd1—O6	69.7 (3)
C6—C5—C8—O2	177.1 (4)	C14—O8—Cd1—O5	−42.8 (4)
C4—C5—C8—O2	−3.9 (6)	C14—O8—Cd1—N1	2.4 (3)
C6—C5—C8—O1	−3.6 (6)	C14—O8—Cd1—N2	−159.4 (3)
C4—C5—C8—O1	175.5 (4)	C9—O5—Cd1—O7	−149.8 (4)
O5—C9—C10—N1	−56.0 (6)	C9—O5—Cd1—O9	−171.7 (4)
O6—C11—C12—N1	−61.9 (5)	C9—O5—Cd1—O10	80.8 (3)
N1—C13—C14—O8	−49.3 (6)	C9—O5—Cd1—O6	−83.1 (3)
O7—C15—C16—N2	−53.9 (5)	C9—O5—Cd1—O8	22.5 (4)
N2—C17—C18—O9	−59.8 (5)	C9—O5—Cd1—N1	−23.1 (3)
N2—C19—C20—O10	−55.9 (6)	C9—O5—Cd1—N2	146.0 (3)
C9—C10—N1—C13	−81.6 (5)	C10—N1—Cd1—O7	48.2 (3)
C9—C10—N1—C12	155.2 (4)	C13—N1—Cd1—O7	168.8 (3)
C9—C10—N1—Cd1	36.4 (5)	C12—N1—Cd1—O7	−71.7 (3)
C14—C13—N1—C10	171.0 (4)	C10—N1—Cd1—O9	161.8 (3)
C14—C13—N1—C12	−67.0 (5)	C13—N1—Cd1—O9	−77.6 (3)
C14—C13—N1—Cd1	50.1 (5)	C12—N1—Cd1—O9	41.9 (3)
C11—C12—N1—C10	−81.3 (5)	C10—N1—Cd1—O10	−80.1 (3)
C11—C12—N1—C13	155.9 (4)	C13—N1—Cd1—O10	40.5 (3)
C11—C12—N1—Cd1	39.7 (4)	C12—N1—Cd1—O10	160.0 (3)
C15—C16—N2—C19	165.7 (4)	C10—N1—Cd1—O6	110.8 (3)
C15—C16—N2—C17	−72.8 (5)	C13—N1—Cd1—O6	−128.6 (3)
C15—C16—N2—Cd1	45.9 (4)	C12—N1—Cd1—O6	−9.1 (3)
C20—C19—N2—C16	−86.5 (5)	C10—N1—Cd1—O8	−146.6 (3)
C20—C19—N2—C17	151.3 (4)	C13—N1—Cd1—O8	−26.0 (3)
C20—C19—N2—Cd1	31.7 (5)	C12—N1—Cd1—O8	93.4 (3)
C18—C17—N2—C16	150.9 (4)	C10—N1—Cd1—O5	−7.6 (3)
C18—C17—N2—C19	−86.7 (5)	C13—N1—Cd1—O5	113.0 (3)
C18—C17—N2—Cd1	33.4 (5)	C12—N1—Cd1—O5	−127.6 (3)
C10—C9—O5—Cd1	49.1 (5)	C10—N1—Cd1—N2	−47.5 (6)
C12—C11—O6—Cd1	51.2 (4)	C13—N1—Cd1—N2	73.1 (5)
C16—C15—O7—Cd1	34.1 (5)	C12—N1—Cd1—N2	−167.5 (4)
C13—C14—O8—Cd1	22.1 (5)	C16—N2—Cd1—O7	−20.5 (3)
C17—C18—O9—Cd1	56.8 (4)	C19—N2—Cd1—O7	−141.1 (3)
C19—C20—O10—Cd1	53.3 (5)	C17—N2—Cd1—O7	99.2 (3)
C15—O7—Cd1—O9	58.4 (3)	C16—N2—Cd1—O9	−123.0 (3)
C15—O7—Cd1—O10	−54.6 (4)	C19—N2—Cd1—O9	116.4 (3)
C15—O7—Cd1—O6	130.1 (3)	C17—N2—Cd1—O9	−3.3 (3)
C15—O7—Cd1—O8	93.1 (6)	C16—N2—Cd1—O10	117.2 (3)
C15—O7—Cd1—O5	−115.5 (3)	C19—N2—Cd1—O10	−3.4 (3)
C15—O7—Cd1—N1	−169.8 (3)	C17—N2—Cd1—O10	−123.1 (3)
C15—O7—Cd1—N2	−7.8 (3)	C16—N2—Cd1—O6	−72.6 (3)
C18—O9—Cd1—O7	−94.5 (3)	C19—N2—Cd1—O6	166.8 (3)
C18—O9—Cd1—O10	29.3 (3)	C17—N2—Cd1—O6	47.1 (3)
C18—O9—Cd1—O6	−167.4 (3)	C16—N2—Cd1—O8	174.4 (3)
C18—O9—Cd1—O8	93.7 (3)	C19—N2—Cd1—O8	53.8 (3)
C18—O9—Cd1—O5	−73.8 (5)	C17—N2—Cd1—O8	−65.9 (3)
C18—O9—Cd1—N1	142.8 (3)	C16—N2—Cd1—O5	45.2 (3)
C18—O9—Cd1—N2	−28.5 (3)	C19—N2—Cd1—O5	−75.4 (3)
C20—O10—Cd1—O7	20.2 (4)	C17—N2—Cd1—O5	164.9 (3)
C20—O10—Cd1—O9	−85.1 (3)	C16—N2—Cd1—N1	82.2 (5)
C20—O10—Cd1—O6	−176.3 (4)	C19—N2—Cd1—N1	−38.4 (6)
C20—O10—Cd1—O8	−151.9 (4)	C17—N2—Cd1—N1	−158.1 (4)

### Hydrogen-bond geometry (Å, º)

**Table:**

*D*—H···*A*	*D*—H	H···*A*	*D*···*A*	*D*—H···*A*
O6—H01···O4	0.84 (2)	1.86 (2)	2.692 (4)	168 (5)
O8—H02···O2^i^	0.83 (2)	1.79 (2)	2.612 (4)	170 (5)
O5—H03···O4^ii^	0.84 (2)	1.82 (2)	2.645 (5)	170 (6)
O9—H04···O1^i^	0.84 (2)	1.84 (2)	2.673 (4)	169 (5)
O10—H05···O1^iii^	0.84 (2)	1.82 (2)	2.647 (4)	169 (6)
O7—H06···O3	0.86 (2)	1.78 (2)	2.635 (4)	171 (5)

Symmetry codes: (i) *x*, −*y*−1/2, *z*−1/2; (ii) −*x*+1/2, *y*+1/2, *z*; (iii) −*x*+1/2, −*y*, *z*−1/2.
