# Supplementary figures and images for: Crystal structure of bis­{2-[bis­(2-hy­droxy­eth­yl)amino]­ethanol-κ4 O,N,O′,O′′}cadmium terephthalate
Source: Acta Crystallogr Sect E Struct Rep Online. 2014 Oct 18;70(Pt 11):m371. doi: 10.1107/S1600536814022375 (PMC4257248; doi:10.1107/S1600536814022375)

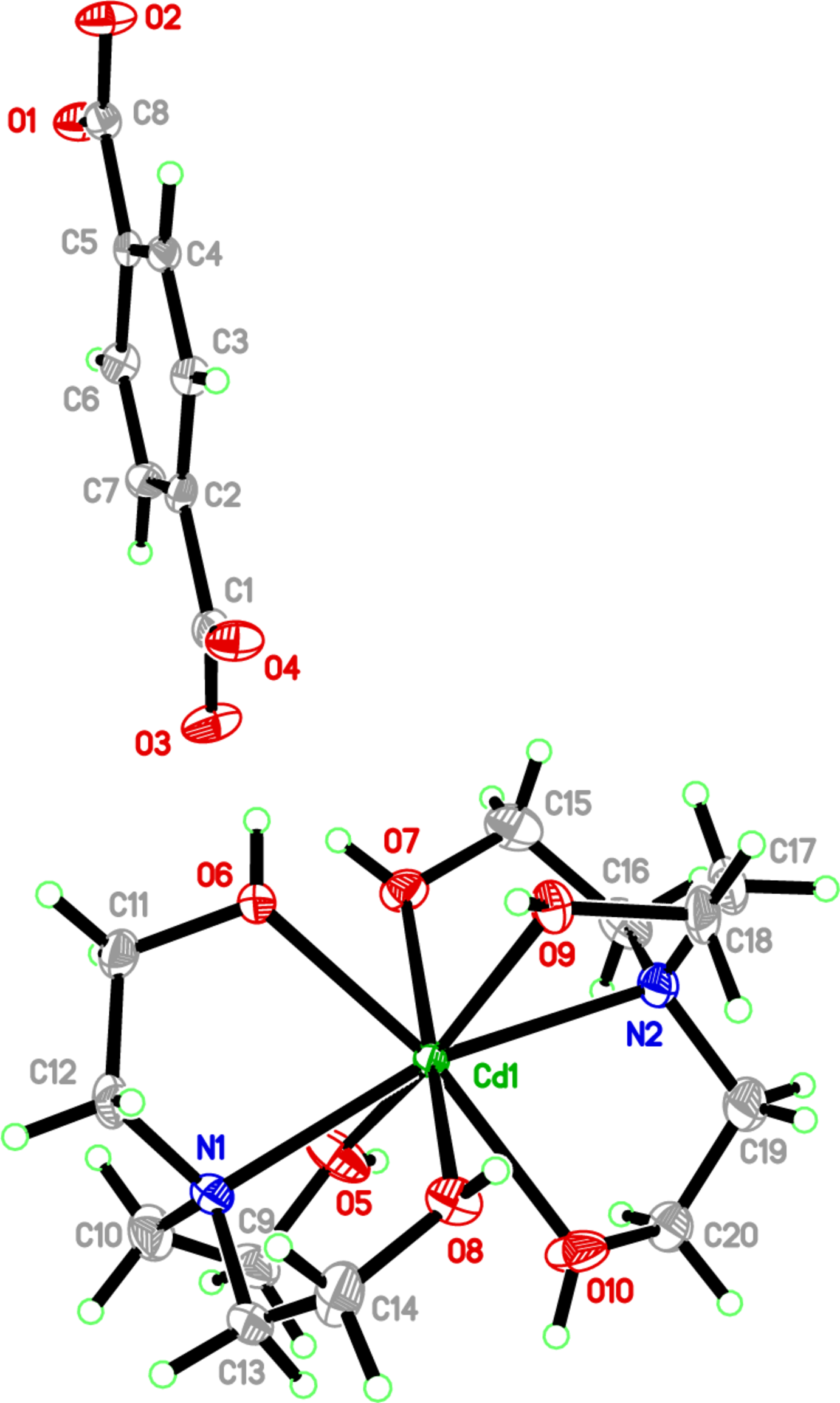

Supplement: Supplementary file 3 [file e-70-0m371-fig1.tif]

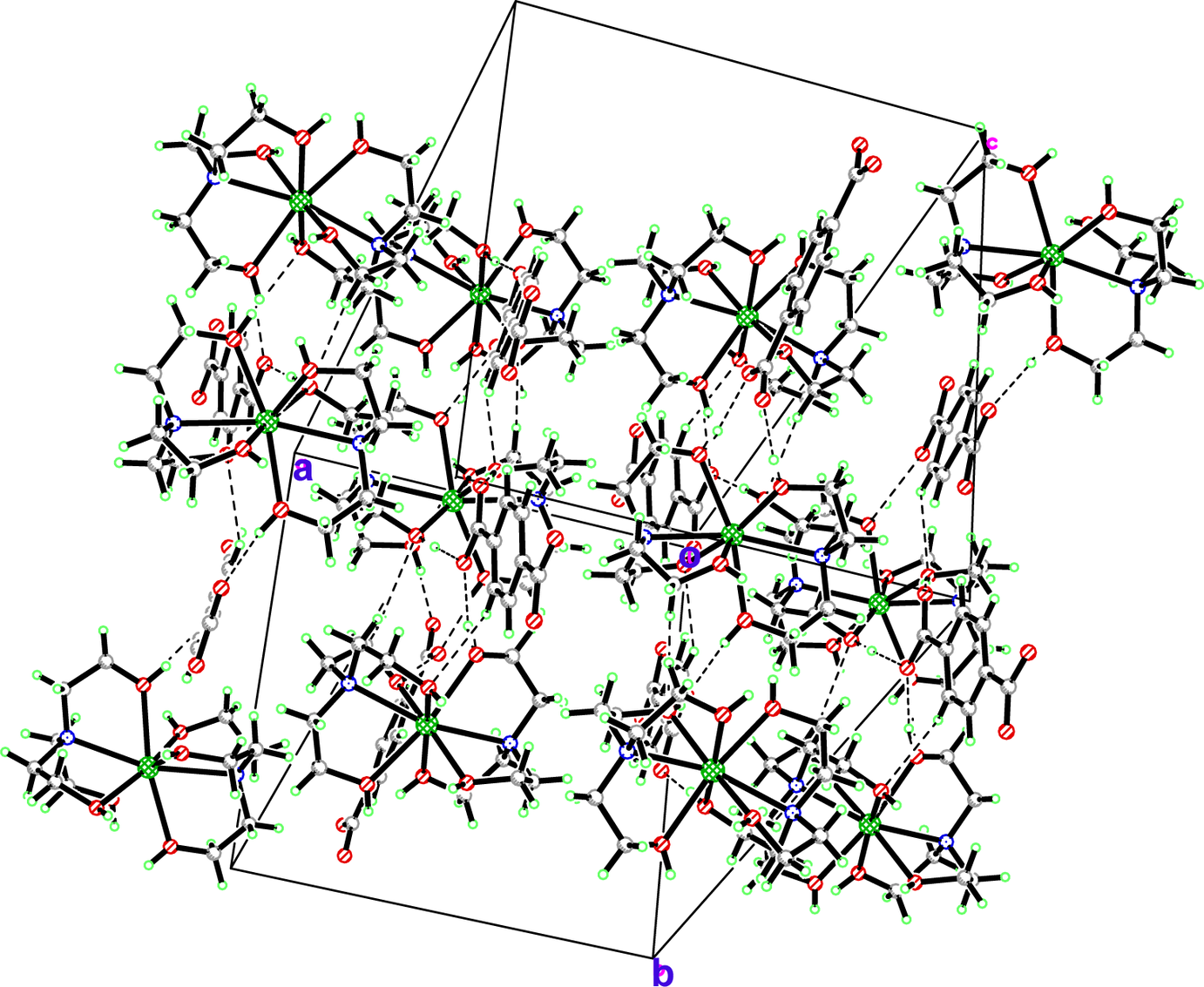

Supplement: Supplementary file 4 [file e-70-0m371-fig2.tif]
